# Sugt1 loss in skeletal muscle stem cells impairs muscle regeneration and causes premature muscle aging

**DOI:** 10.1093/lifemedi/lnad039

**Published:** 2023-11-02

**Authors:** Zhiming He, Xiaona Chen, Gexin Liu, Yuying Li, Feng Yang, Hao Sun, Huating Wang

**Affiliations:** Department of Chemical Pathology, Li Ka Shing Institute of Health Sciences, The Chinese University of Hong Kong, Hong Kong 999077, China; Center for Neuromusculoskeletal Restorative Medicine (CNRM), CUHK InnoHK Centres, The Chinese University of Hong Kong, Hong Kong 999077, China; Department of Orthopaedics and Traumatology, Li Ka Shing Institute of Health Sciences, The Chinese University of Hong Kong, Hong Kong 999077, China; Department of Orthopaedics and Traumatology, Li Ka Shing Institute of Health Sciences, The Chinese University of Hong Kong, Hong Kong 999077, China; Department of Orthopaedics and Traumatology, Li Ka Shing Institute of Health Sciences, The Chinese University of Hong Kong, Hong Kong 999077, China; Department of Chemical Pathology, Li Ka Shing Institute of Health Sciences, The Chinese University of Hong Kong, Hong Kong 999077, China; Department of Chemical Pathology, Li Ka Shing Institute of Health Sciences, The Chinese University of Hong Kong, Hong Kong 999077, China; Center for Neuromusculoskeletal Restorative Medicine (CNRM), CUHK InnoHK Centres, The Chinese University of Hong Kong, Hong Kong 999077, China; Department of Orthopaedics and Traumatology, Li Ka Shing Institute of Health Sciences, The Chinese University of Hong Kong, Hong Kong 999077, China

**Keywords:** muscle stem cell, Sugt1, cellular senescence, aging, Trim21

## Abstract

Adult skeletal muscle stem cells (MuSCs) are essential for muscle homeostasis and regeneration. During aging, the number of MuSCs and their regenerative capacity gradually decline but the underlying mechanisms remain elusive. Here, we identify Sugt1 (suppressor of G2 allele of SKP1 homolog), which is a chaperone for kinetochore function during mitosis and is essential for muscle regeneration by regulating MuSC proliferation. Sugt1 expression level is low in quiescent MuSCs but highly induced when the cells become activated and expand as proliferating myoblasts. Inducible inactivation of Sugt1 in MuSCs causes impaired muscle regeneration upon acute injury by impairing MuSC proliferation. Furthermore, loss of Sugt1 leads to cell cycle arrest in the G2/M phase and cellular senescence. Moreover, long-term loss of Sugt1 in MuSCs results in precocious muscle aging by inhibiting MuSC cell proliferation and promoting cellular senescence. Mechanistically, we identify a cytosolic E3 ubiquitin-ligase, Trim21 as a protein interacting partner for Sugt1 in myoblast cells. We further demonstrate that Sugt1 promotes the ubiquitination of p21 via Trim21; and Sugt1 loss causes p21 accumulation to inhibit cell cycle progression and stimulates cellular senescence. Collectively, our findings uncover that Sugt1 is an essential regulator for MuSC regenerative function during muscle regeneration and aging.

## Introduction

Skeletal muscle has a remarkably robust regenerative capacity which largely relies on muscle stem cells (MuSCs), also known as muscle satellite cells [1]. Under normal conditions, MuSCs are located in a niche beneath the basal lamina of muscle fibers and maintain their quiescent status [2]. Upon muscle injury, MuSCs are immediately activated and rapidly undergo proliferative expansion as myoblasts, followed by differentiation to form myotubes and fusion to form new myofibers, repairing the muscle damage [3, 4]. A subset of activated MuSCs (ASCs) return to quiescence to maintain the stem cell pool [3, 5].

Aging is becoming a global healthcare challenge and skeletal muscle declines in muscle mass, function, and regenerating capacity, contributing to the pathogenesis of aging-related diseases, e.g. sarcopenia [6, 7]. Cellular senescence is an important mechanism underlying tissue aging and is characterized by a permanent arrest in the cell cycle and loss of the ability to divide and proliferate [8, 9]. Senescent cells also specifically express marker genes such as p16, p21, and SA-β-gal, etc. [10, 11]. Recently, accumulating evidence shows that cellular senescence of MuSCs, which results in dysfunction in stem cell pool self-renewal and reduction of regenerative capacity, is closely associated with sarcopenia upon aging [9, 12, 13]. However, the molecular regulatory mechanism for MuSC senescence is still largely unknown.

Sugt1, a suppressor of the G2 allele of SKP1 homolog, is a multifunctional protein and a co-chaperone protein that functions together with heat shock protein 90 (HSP90) to regulate cell cycle progression by mediating kinetochore complex assembly in yeast and human [14–17]. *SUGT1* depletion in HeLa cells can induce irregular chromosome alignment and cause cell cycle arrest in mitosis due to activating the spindle assembly checkpoint [14, 17, 18]. Sugt1 binds to HSP90 to activate the SCF ubiquitin ligase complex and CBF3 kinetochore complex through interacting with SKP1 protein [16, 19]. Furthermore, PLK1-mediated phosphorylation at Ser331 of Sugt1 recruits Sugt1 to the kinetochore and stabilizes the MIS12 complex by enhancing the interaction of Sugt1 to DSN1 [20]. Notably, Jia et al. demonstrated that PLK1 deletion severely affects the regenerative function of MuSCs [21]. The Sugt1-TPR domain is also reported to interact with the D2 and D3 domains of eEF1A1, hinting that Sugt1 may compete with viral RNA in the interaction of eEF1A1 [22]. In humans, the Sugt1-SGS domain is implicated to interact with the calcyclin S100 protein family, such as S100A6, in a calcium-dependent manner [23]. Our recent study demonstrated that Sugt1-associated muscle-long noncoding RNA *SAM* regulates MuSC proliferation through stabilizing Sugt1 and facilitating kinetochore assembly in myoblast cells [24]. Knocking down Sugt1 in C2C12 cells and MuSCs suppresses cell proliferation, suggesting that Sugt1 plays an important role in regulating MuSC functions.

To further elucidate Sugt1 function in regulating MuSC and muscle regeneration, in this study, we reveal that inactivation of Sugt1 in MuSCs results in impaired muscle regeneration by inhibiting MuSC proliferation and increasing cellular senescence. Moreover, Sugt1 loss leads to premature aging of the mice with aggravated sarcopenia progression. Mechanistically we reveal that Sugt1 interacts with Trim21 to facilitate Trim21-mediated p21 degradation, thus promoting MuSC proliferation and inhibiting cellular senescence. Altogether, our findings uncover the functional and mechanistic role of Sugt1 in MuSCs during regeneration and aging, indicating that Sugt1 can be a potential therapeutic target for sarcopenia treatment.

## Results

### Ablation of Sugt1 in adult MuSCs leads to delayed muscle regeneration

**Figure 1.**
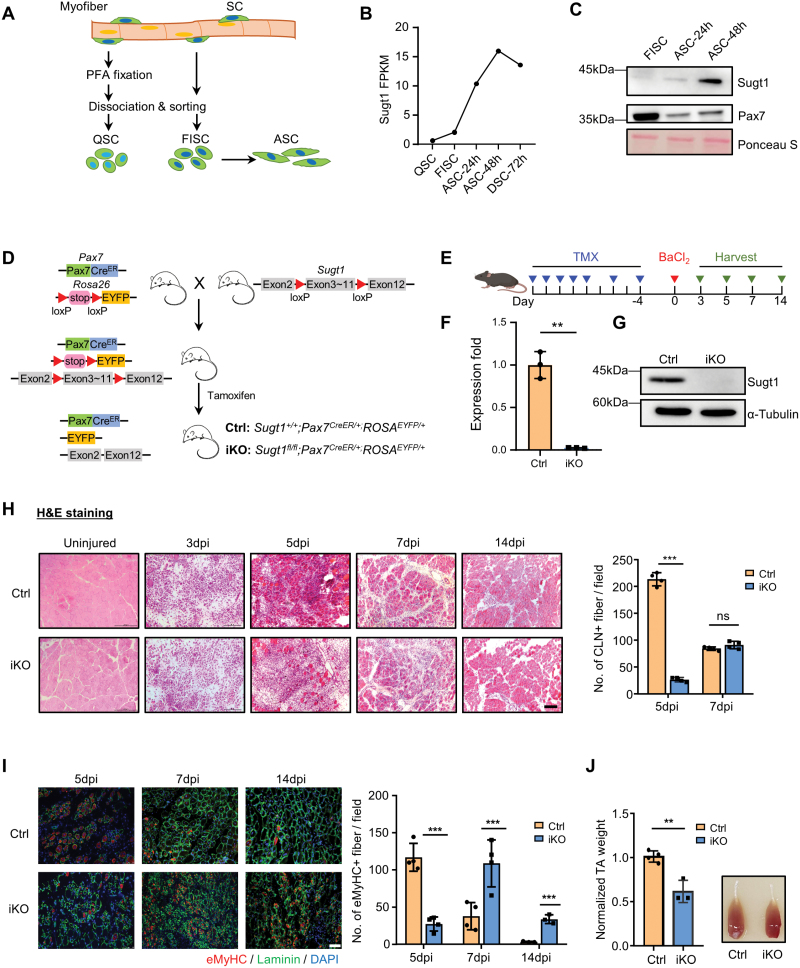
**Ablation of Sugt1 in MuSC leads to impaired muscle regeneration.**(A) Schematic illustration of satellite cells collection from *Pax7-nGFP* mice. QSC: pre-fixed by PFA. FISC: freshly isolated SCs. ASC: activated SCs in culture. (B) The expression level of Sugt1 mRNA from analyzing the RNA-seq data of the above cells. (C) The expression level of Sugt1 protein was probed by Western blotting in FISC and ASC. Ponceau S staining was used as the loading control. (D) Schematic illustration of the strategy to inactivate Sugt1 in inducible knockout (iKO) mice. *Sugt1*^*flox/flox*^ mice were mated with *Pax7*^*CreER*^*/ ROSA*^*EYFP*^ mice; the exons between exon 3 to exon 11 of *Sugt1* were deleted in the iKO mice after TMX injection which also resulted in the removal of the stop signal of YFP at the *Rosa26* site to allow the expression of YFP in iKO MuSCs. (E) Schematic illustration of seven doses of TMX injection to delete *Sugt1* in the iKO mouse. BaCl_2_ was injected into TA muscles of the above Ctrl or iKO mice 4 days post-TMX injection to induce acute injury. The injected TA muscles were harvested at the designated times for the assessment of the regeneration process. (F) Loss of Sugt1 was confirmed by qRT-PCR with 18S rRNA used for normalization. (G) Loss of Sugt1 was confirmed by Western blotting with α-Tubulin as the loading control. (H) Left: H&E staining of the above collected TA muscles at designated dpi. Scale bar: 100 μm. Right: Quantification of the CLN fibers per fiber at 5 and 7 dpi (*n* = 4 mice). (I) Left: IF staining of eMyHC and laminin on the above TA muscle at 5, 7, 14 dpi, Scale bar: 50 μm. Right: Quantification of the number of eMyHC positive fibers per field. *n* = 4 mice. (J) Left: TA muscle weight was quantified at 14 dpi and normalized to the Ctrl. *n* = 3 mice. Right: Representative image of the above TA muscles data are represented as mean ± SD (standard deviation). Statistical significance was determined using a two-tailed Student’s *t* test.

To dissect the functional role of Sugt1 in MuSCs and muscle regeneration, we first examined the expression dynamic of Sugt1 in MuSCs prepared from limb muscles of *Pax7-nGFP* mice [25]. Quiescent cells (QSCs) were obtained by performing *in situ* paraformaldehyde (PFA) fixation before fluorescence-activated cell sorting (FACS) which has been proven to preserve the quiescence state of MuSCs [26, 27]. Freshly isolated cells (FISCs) were cultured with growth medium for 24 h to become activated (ASC-24h), which were further cultured to proliferate (ASC-48h) and differentiate (DSC-72h) (Fig. 1A). By analyzing RNA sequencing (RNA-seq) data available from the above cells using our recently generated RNA-seq datasets [28], we found that Sugt1 was sharply up-regulated (5.02-fold) in ASC-48h vs. FISC, but down-regulated (15.05%) in DSC-72h vs. ASC-48h (Fig. 1B). Consistently, the protein level of Sugt1 was slightly up-regulated in ASC-24h and sharply increased in ASC-48h when MuSCs were undergoing intensive proliferation (Fig. 1C). Moreover, cellular fractionation assay revealed that Sugt1 proteins were mainly distributed in the cytoplasmic fraction of C2C12 myoblasts (Fig. S1A). Additionally, we found that Sugt1 was highly expressed in G1 phase and downregulated in S phase and G2/M phase of the cell cycle (Fig. S1B). These results indicate that Sugt1 expression is highly induced in activating and proliferating MuSCs, suggesting its potential function in regulating MuSC proliferation and muscle regeneration.

To test the above notion, we generated an inducible Sugt1 knockout mouse strain by crossing *Sugt1*^*flox/flox*^ mouse [24] with the *Pax7*^*CreER*^*/ROSA*^*EYFP*^ allele [25, 29] to specifically inactivate Sugt1 in MuSCs (Fig. 1D). Genetic deletion of Sugt1 in MuSCs was induced by intraperitoneal injection (i.p.) of tamoxifen (TMX) in adult (2-month-old) mice (Fig. 1E) and a successful deletion (97.6%) of Sugt1 mRNA (Fig. 1F) and protein (Fig. 1G) level was detected in the Sugt1 inducible knockout (iKO) MuSCs as compared to the control (Ctrl) littermate. The iKO mice did not show obvious morphology changes with comparable body size and weight with the Ctrl littermates (Fig. S1C and S1D).

Next to investigate whether the Sugt1 ablation affects muscle regeneration, acute muscle injury was induced by Barium Chloride (BaCl_2_) injection into the tibialis anterior (TA) muscle of both adult Ctrl and iKO mice. After the injury, muscle tissues undergo degeneration characterized by intensive myofiber necrosis and inflammatory cells infiltration in the first 2 days and muscle regeneration can be observed at 5 days post-injury (dpi) featured by the appearance of newly formed fibers with centrally localized nuclei (CLN) and expression of embryonic myosin heavy chain (eMyHC). Full muscle reconstruction is completed by 4 weeks after injury. Respectively, we collected the TA muscle from Ctrl and iKO mice at 3, 5, 7, and 14 dpi to evaluate muscle regeneration progression. H&E staining showed that the number of fibers with CLN per field was significantly decreased (87.7%) in iKO vs. Ctrl mice at 5 dpi (Fig. 1H); Consistently, the immunofluorescent (IF) staining of eMyHC showed that the number of eMyHC+ fibers was also decreased by 76.5% at 5 dpi (Fig. 1I). However, the number of eMyHC+ fibers significantly increased in iKO muscles at 7 and 14 dpi (65.2% and 90.1%, respectively), indicating delayed muscle regeneration in the iKO muscles (Fig. 1I). At 14 dpi, the TA muscle mass was decreased (39.4%) in iKO vs. Ctrl (Fig. 1J). Altogether, these results demonstrate that loss of Sugt1 in MuSCs causes a marked delay of muscle regeneration after acute injury; thus, Sugt1 is required for this process.

### Deletion of Sugt1 in MuSCs leads to cell autonomous defects in proliferation and promotes cellular senescence

**Figure 2.**
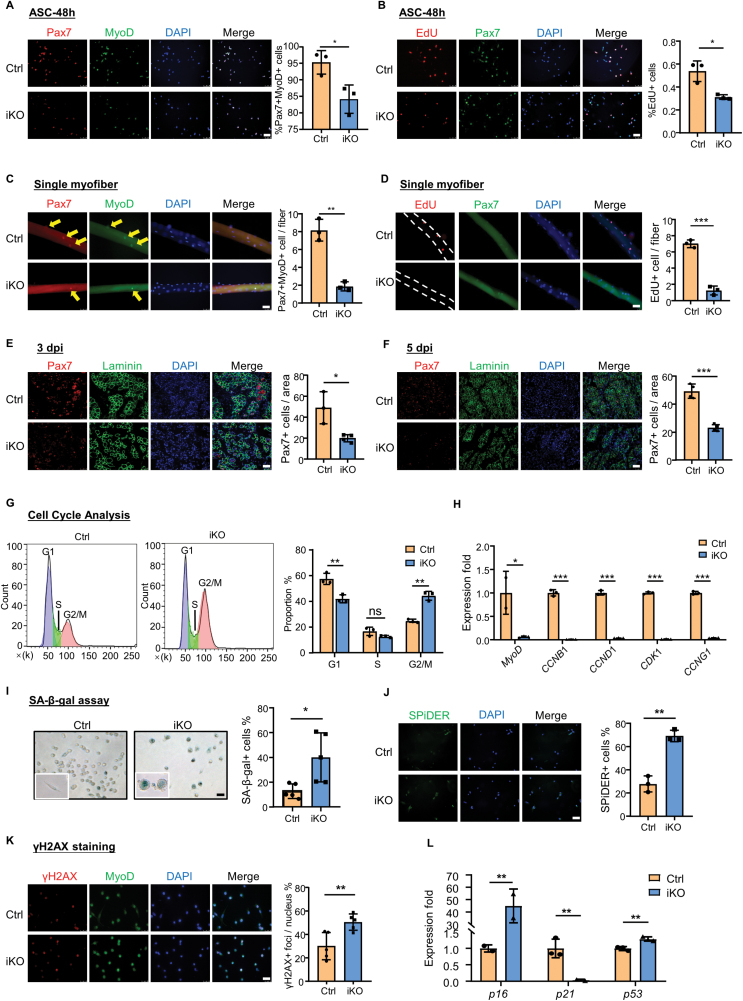
**Deletion of Sugt1 in MuSCs leads to cell autonomous defects in proliferation and promotes cellular senescence.**(A) Left: IF staining for Pax7 and MyoD were performed in ASCs isolated from Ctrl or iKO mice and cultured for 48 h; Scale bar: 50 μm. Right: Quantification of the percentage of Pax7+MyoD+ cells. *n* = 3 mice. (B) Left: EdU incorporation assay was performed and IF staining for Pax7 and EdU was performed in ASC-48h. Scale bar: 50 μm. Right: Quantification of the percentage of Pax7+EdU+ cells. *n* = 3 mice. (C) Left: single myofibers were isolated from Ctrl or iKO mice and IF staining for Pax7 and MyoD was performed at 48 h. Scale bar: 50 μm; Right: Quantification of the number of Pax7+MyoD+ cells per myofiber. *n *= 3 mice, > 30 myofibers were counted for each mouse. (D) Left: single myofibers were isolated from Ctrl or iKO mice and EdU incorporation assay was performed and IF staining for Pax7 and EdU was performed at 48 h. Scale bar: 50 μm; Right: Quantification of the number of Pax7+EdU+ per myofiber (*n *= 3 mice, over 30 myofibers were counted for each mouse). (E) Left: TA muscles were dissected from Ctrl or iKO mice and IF staining for Pax7 and laminin was performed at 3 dpi. Scale bar: 50 μm. Right: Quantification of the number of Pax7+ cells per section area. *n* = 3 mice. (F) Left: TA muscles were dissected from Ctrl or iKO mice and IF staining for Pax7 and laminin was performed at 5 dpi. Scale bar: 50 μm. Right: Quantification of the number of Pax7+ cells per section area. *n* = 3 mice. (G) Left: Cell cycle analysis was performed on MuSCs isolated from Ctrl or iKO mice and cultured for 48 h; Right: The distribution of the percentage of cells at each phase in MuSCs. (H) MuSCs from Ctrl or Sugt1 iKO mice were isolated and cultured for 2.5 days; The expression of the indicated genes was detected by qRT-PCR. (I) Left: SA-β-gal staining was performed for MuSCs cultured for 5 days. Scale bar: 50 μm; Right: Quantification of the percentage of SA-β-gal+ cells. *n* = 5 mice. (J) Left: SPiDER β-gal fluorescence staining was performed on MuSCs cultured for 72 h. Scale bar: 50 μm. Right: Quantification of the percentage of SPiDER+ cells. *n *= 3 mice. (K) Left: IF staining for γH2AX and MyoD was performed on MuSCs cultured for 72 h. Scale bar: 50 μm; Right: Quantification of the percentage of γH2AX+ foci. *n* = 5 mice. (L) qRT-PCR quantification of the indicated gene in Ctrl or iKO MuSCs cultured for 72 h. *n* = 3 mice. Data are represented as mean ± SD. Statistical significance was determined using a two-tailed Student’s *t* test.

To pinpoint the defects of MuSCs upon Sugt1 deletion, we first assessed the proliferative capacity of iKO and Ctrl cells. MuSCs isolated from iKO, or Ctrl mice were cultured for 2 days, and IF staining of Pax7 and MyoD showed a significant reduction of the percentage of Pax7+MyoD+ cells in iKO (84.14%) vs. Ctrl (95.48%) (Fig. 2A). Moreover, EdU incorporation assay was performed, and we observed a significant decrease of the percentage of EdU+ cells in iKO (31.23%) vs. Ctrl (53.78%) (Fig. 2B). Consistently, similar results were obtained when we performed the above assays on isolated single myofibers from Ctrl or iKO mice. A decreased number of Pax7+MyoD+ cells (by 76.99% decrease) (Fig. 2C) or EdU+ (by 82.23% decrease) cells per myofiber was observed in iKO vs. Ctrl muscles (Fig. 2D). Moreover, we performed the overexpression assay by transfecting a Flag-tagged Sugt1 expressing plasmid in C2C12 myoblast cell line and found the percentage of EdU+ cells significantly increased (by 70.99%) upon Sugt1 overexpression (Fig. S2A). Consistently, in response to muscle injury, the number of Pax7+ cells was also remarkably reduced in the iKO muscle compared with Ctrl at both 3 dpi and 5dpi (59.4% and 53.1%, respectively) (Fig. 2E and 2F). Altogether, these results demonstrate that ablation of Sugt1 impairs SC proliferation both *in vitro* and *in vivo* during injury-induced muscle regeneration.

In addition, we also examined if Sugt1 ablation affects MuSC activation at 24 h in culture and found no significant difference in the percentage of EdU+ or Pax7+MyoD+ cells in iKO vs. WT (Fig. S2B and S2C). The observation was also substantiated on single myofibers (Fig. S2D), altogether indicating that Sugt1 ablation may not impact the activation of SC. Interestingly, when the differentiation was examined, we found a significant reduction of the percentage of MyoG+MyoD+ cells in iKO (69.63%) compared to WT cells (85.27%) cultured for 72 h to differentiate (DSCs) (Fig. S2E), Consistently, the number of MyoG+MyoD+ cells was also decreased (by 18.33%) on myofibers isolated from iKO and cultured for 72 h (Fig. S2F). Moreover, we observed a slightly decreased fusion capacity in iKO vs. WT DSCs by MF20 staining (Fig. S2G). Altogether, the above findings indicate Sugt1 deletion may hinder the differentiation of myoblasts.

Sugt1 is known to regulate cell mitosis; therefore, we further investigated whether the attenuated SC proliferation upon Sugt1 loss was due to abnormal cell cycle progression. Cell cycle analysis was performed on MuSCs cultured for 48 h and revealed that Sugt1 deletion caused myoblast cell cycle arrest at G2/M phase transition (Fig. 2G). Moreover, RT-qPCR revealed that expression levels of cell cycle genes, including *CCNB1*, *CCND1*, *CDK1*, and *CCNG1* were dramatically down-regulated in iKO cells cultured for 2.5 days (Fig. 2H), demonstrating that the proliferative defect of iKO cells may be due to cell cycle arrest induced by Sugt1 deletion.

It is known that inhibited proliferation and cell cycle arrest can cause cellular senescence. We next cultured MuSCs from Ctrl or iKO mice for 5 days and examined cellular senescence. Staining of SA-β-gal, a commonly used senescence marker, revealed that the percentage of SA-β-gal+ cells was significantly increased in iKO (39.98%) vs. Ctrl (12.90%) cells (Fig. 2I). Furthermore, staining of a more sensitive fluorescent SA-β-gal detection probe SPiDER-β-gal was performed in cells cultured for 72 h and a significant increase (by 59.9%) in the percentage of SPiDER-β-gal+ cells was detected in iKO vs. Ctrl cells (Fig. 2J), suggesting Sugt1 loss leads to increased myoblast senescence. This was further confirmed by IF staining of histone γH2AX, another commonly used marker of cellular senescence that is associated with DNA double-strand breaks and a significant increase (by 68.20%) in the percentage of γH2AX foci+ cells was detected in iKO vs. Ctrl (Fig. 2K). Moreover, qRT-PCR detection showed that the expression levels of *p16* and *p53* were also significantly up-regulated (44.86-fold) in iKO vs. Ctrl cells (Fig. 2L). Altogether, these results demonstrate that loss of Sugt1 causes a cell-autonomous defect in MuSCs proliferation and results in precocious cellular senescence.

### Long-term loss of Sugt1 results in precocious muscle aging by reducing MuSC regenerative capacity and promoting MuSC senescence

**Figure 3.**
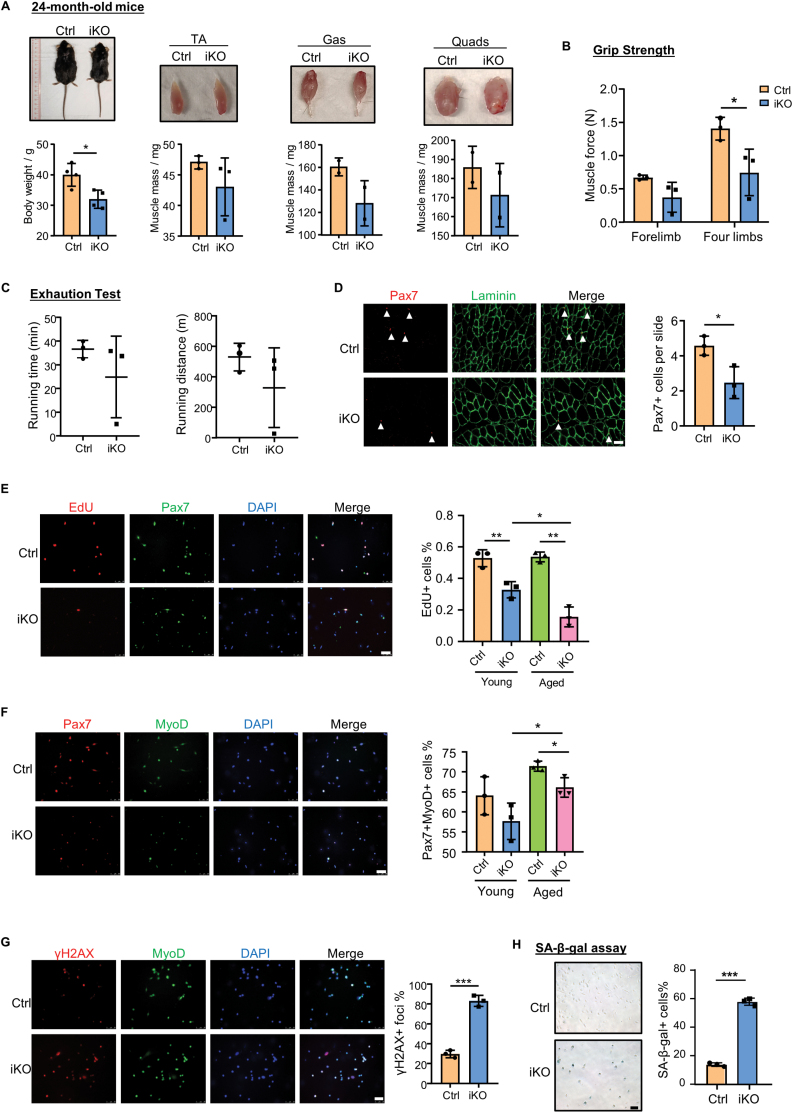
**Long-term loss of Sugt1 Results in precocious muscle aging by reducing MuSC regenerative capacity and promoting MuSC senescence.**(A) Top: Representative whole body and muscle images of 24-month-old Ctrl and iKO mice. TA: tibialis anterior muscle, Gas: gastrocnemius muscle, and Quads: quadriceps extensor muscle. Bottom: Quantification of body weight and muscle mass. *n* = 3 mice. (B) Grip strength was measured in the forelimb and four limbs of aged Ctrl and iKO mice; each mouse was tested 5 times, and the mean was presented. *n* = 3 mice. (C) Aged Ctrl or iKO mice were subject to a treadmill running exhaustion test and the total running time/distance was recorded. *n* = 3 mice. (D) Left: IF staining for Pax7 and Laminin in TA muscles of aged Ctrl and iKO mice. Sale bar: 50 μm. Right: Quantification of the number of Pax7+ cells. *n *= 3 mice per group. (E) Left: EdU incorporation assay was performed and IF staining for Pax7 and EdU was performed in ASC-48h. Scale bar: 50 μm; Right: Quantification of the percentage of Pax7+EdU+ cells. *n* = 3 mice. (F) Left: IF staining for Pax7 and MyoD was performed in ASCs isolated from 24-month-old Ctrl or iKO mice and cultured for 48 h; Scale bar: 50 μm. Right: Quantification of the percentage of Pax7+MyoD+ cells. *n* = 3 mice. (G) Left: IF staining for γH2AX and MyoD was performed in MuSCs isolated from 24-month-old Ctrl or iKO mice and cultured for 72 h; Scale bar: 50 μm; Right: Quantification of the percentage of γH2AX+MyoD+ cells (*n* = 3 mice). (H) Left: SA-β-gal staining was performed for MuSCs cultured for 5 days. Scale bar: 50 μm; Right: Quantification of the percentage of SA-β-gal+ cells. *n* = 5 mice. Data are represented as mean ± SD for all graphs. Statistical significance was determined using a two-tailed Student’s *t* test or one-way ANOVA.

Cellular senescence of MuSCs is closely associated with muscle aging and sarcopenia condition [8, 12]. Therefore, we next examined the long-term effect of Sugt1 deletion in MuSCs and skeletal muscle during the natural aging process. After injecting with TMX, 2-month-old Ctrl and iKO mice were house held until 24 months old. We observed a significant decrease in body size and body weight in aged iKO mice compared to Ctrl (Fig. 3A). Consistently, the size and mass of multiple muscles including TA, Gas (gastrocnemius), and Quads (quadriceps) were also decreased in the iKO mice (Fig. 3A).

To examine whether the muscle function was also affected by Sugt1 inactivation, grip strength [30] was measured and found to be significantly decreased in the forelimb (by 44.4% decrease) as well as the entire four limbs (by 46.9% decrease) of aged iKO vs. Ctrl mice (Fig. 3B). Consistently, when the treadmill running exhausting test was performed [30], the total running time and distance of aged iKO mice showed a modest but not statistically significant reduction (32.1% and 37.9%, respectively) (Fig. 3C). Furthermore, IF staining of Pax7 of the TA muscle sections showed that the number of MuSCs was significantly decreased (by 45.9%) in the aged iKO vs. Ctrl mice (Fig. 3D). Altogether, the above results demonstrate that loss of Sugt1 results in a precocious MuSC pool reduction and muscle aging.

To further investigate the impact of long-term loss of Sugt1 in MuSC function during aging, MuSCs were isolated from 24-month-old iKO and Ctrl mice for EdU incorporation assay. Aged iKO cells showed a significant decline (by 71.0% decrease) in the proliferative ability as compared with the Ctrl cells (Fig. 3E). Interestingly, no significant difference in EdU incorporation was observed between aged and young Ctrl cells while the percentage of EdU+ cells was largely reduced in aged iKO cells vs. young iKO cells. The above results thus demonstrate that Sugt1 loss leads to decreased MuSC proliferative ability. This was further validated by quantifying the percentage of Pax7+MyoD+ cells in aged iKO vs. Ctrl mice (Fig. 3F). Moreover, the percentage of γH2AX+ cells was markedly increased (2.8-fold) in aged iKO vs. Ctrl cells when cultured for 48 h (Fig. 3G), and a higher percentage (57.77%) of SA-β-gal+ cells was also observed when the cells were cultured for 3 days (Fig. 3H), confirming that Sugt1 loss aggravates MuSC senescence during aging. Lastly, when RNA-seq (Table S1) was performed to analyze transcriptomic changes in aged iKO vs. Ctrl MuSCs (Fig. S3A), *p16* was found to be upregulated (Fig. S3B) in the iKO and enrichment of senescence-associated secretory phenotype (SASP) genes was also detected (Fig. S3C). Altogether, these results demonstrate that loss of Sugt1 in MuSCs causes premature muscle aging possibly by decreasing MuSC number and regenerative capacity while promoting MuSC senescence.

### IP/MS analysis identifies Sugt1 protein interactome in myoblasts cells

**Figure 4.**
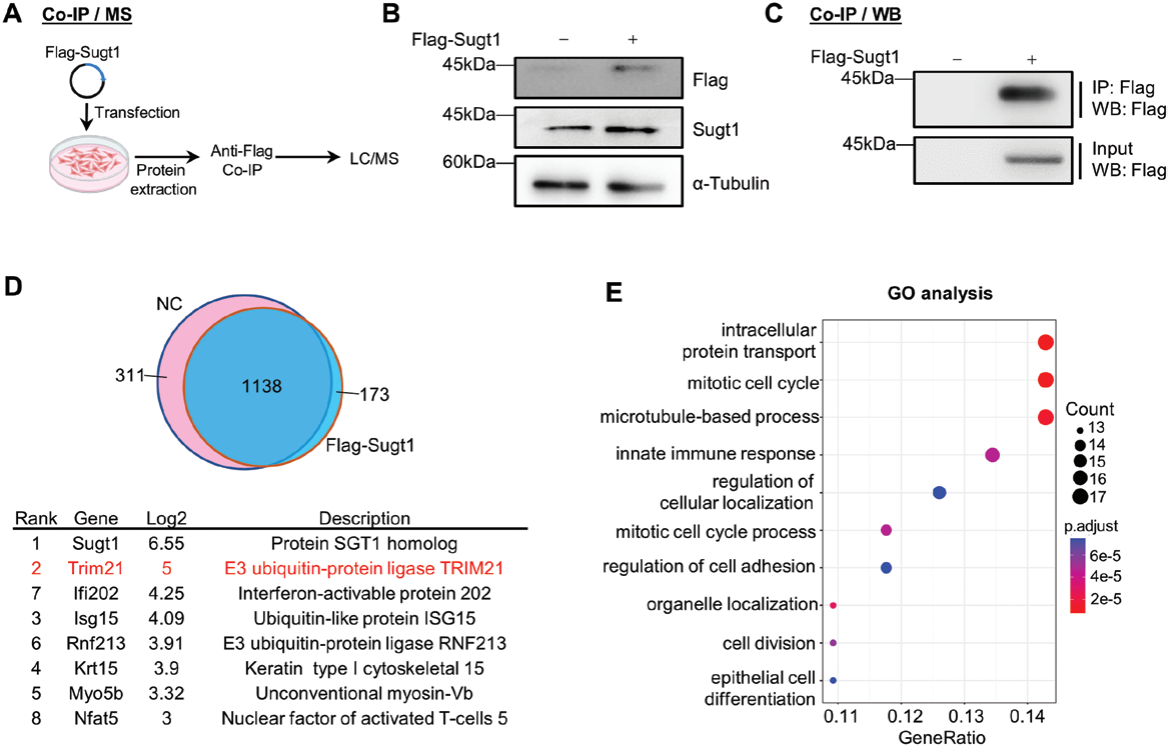
**IP/MS analysis identifies Sugt1 interactome in myoblast cells.**(A) Schematic illustration of the workflow for identifying Sugt1 interactome in C2C12 myoblasts. An empty vector or Flag-tagged Sugt1 plasmid was expressed in C2C12 myoblasts followed by pull-down with Flag beads; the retrieved proteins were subjected to MS with Bruker timsTOF pro. (B) The above Flag-Sugt1 overexpression in C2C12 myoblasts was confirmed by Western blotting using Flag or Sugt1 antibody. α-Tubulin was used as the loading control. (C) The above IP efficiency of the anti-Flag magnetic beads was confirmed by Western blotting using Flag antibody. (D) Top: The retrieved NC and Flag-Sugt1 were compared, and the Venn diagram showed the overlapping. Bottom: The top-ranked proteins enriched by Flag-Sugt1 are shown. Proteins were ranked based on log_2_ fold change comparing spectral peptide counts in Flag-Sugt1 vs. NC. (E) GO analysis for the enriched proteins by Flag-Sugt1.

Since Sugt1 is a cochaperone protein that functions through interacting with other protein targets, to uncover the underlying mechanisms of Sugt1 regulating MuSC proliferation, we adopted immunoprecipitation (IP) followed by mass spectrometry (MS) to identify the protein interactome of Sugt1 in C2C12 myoblast cells. C2C12 myoblasts were overexpressed with a Flag-tagged Sugt1 expressing plasmid and subject to IP with the Flag antibody. The retrieved proteins were then subject to LC/MS proteomic analysis (Fig. 4A). C2C12 cells transfected with an empty pcDNA3.1 (+) vector was used as the negative control (NC). Successful overexpression of Flag-Sugt1 and IP retrieval of Flag-Sugt1 were determined by Western blotting (Fig. 4B and 4C).

The MS data showed that 173 and 311 proteins were uniquely retrieved from the Flag-Sugt1 and NC cells and 1,138 proteins were shared (Fig. 4D). Gene Ontology (GO) analysis revealed that the retrieved Sugt1 binding proteins were enriched for GO terms including “intracellular protein transport”, “microtubule-based process”, “mitotic cell cycle”, “innate immune response”, etc. (Fig. 4E), which was consistent with the known function of Sugt1 [16, 31].

Among these retrieved proteins, Trim21, an E3 ubiquitin-protein ligase, was the highest-ranking protein on the list next to Sugt1 itself (Fig. 4D). Of note, Trim21 has been shown to regulate cell cycle progression and cyclin-dependent kinase inhibitor ubiquitination [32, 33], which is closely correlated with Sugt1 functions, making it a potential candidate for mediating Sugt1 function in myoblast cells.

### Sugt1 binds with Trim21 to regulate p21 ubiquitination in myoblasts

**Figure 5.**
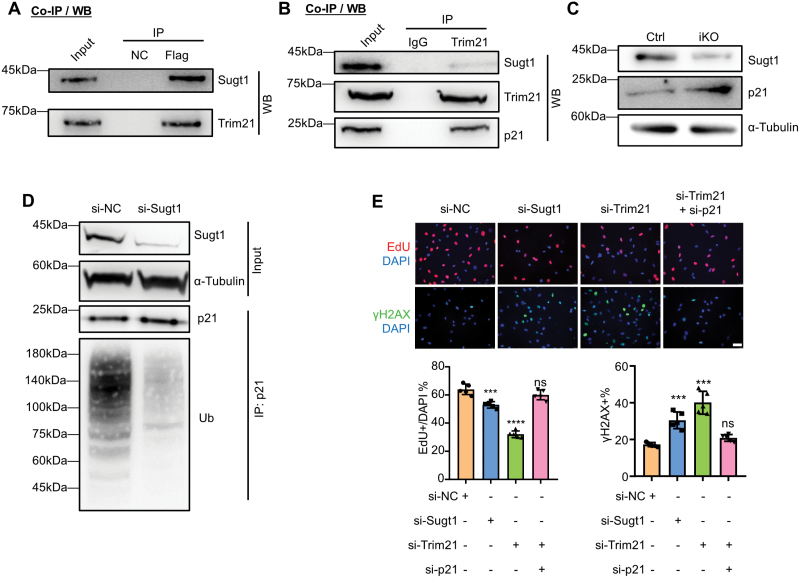
**Sugt1 binds with Trim21 to regulate p21 ubiquitination in myoblasts.**(A–B). Co-IP/Western blotting analysis to confirm the interaction between Trim21 and Sugt1; (A) Flag-tagged Sugt1 was over-expressed in C2C12, and Flag-beads-based IP was performed and followed by Western blotting to verify the retrieved Trim21 protein. (B) IP of endogenous Trim21 protein in C2C12 myoblasts followed by Western blotting to detect the retrieved Sugt1 protein. (C) p21 protein expression level in Sugt1 Ctrl or iKO cells cultured for 3 days were examined by Western blotting with α-Tubulin as the loading control. (D) C2C12 cells were transfected with si-NC or si-Sugt1 oligos and IP was performed with p21 antibody and followed by Western blotting detection of p21 ubiquitination with α-Tubulin as the loading control. (E) Top: C2C12 myoblasts were transfected with the indicated siRNA oligos and after 48 h, EdU incorporation assay or IF staining of γH2AX was performed. Scale bar: 50 μm. Bottom: Quantification of the percentage of EdU+ or γH2AX+ foci. *n* = 5 mice. Data are represented as mean ± SD. Statistical significance was determined using a two-tailed Student’s *t*-test and one-way ANOVA.

To test the functional link between Sugt1 and Trim21, we realized that Trim21 has been reported to catalyze p21 ubiquitination and degradation with its E3 ubiquitin ligase activity [32, 33] and Trim21 deficiency is highly related to tumor cell proliferation and cellular senescence [33]. We first confirmed the interaction of Sugt1 and Trim21 with co-immunoprecipitation (co-IP) assay using antibodies against Flag-Sugt1 or Trim21 in C2C12 myoblasts and the result showed evident interaction between Sugt1 and Trim21 proteins (Fig. 5A and 5B). Furthermore, co-IP assay using Trim21 antibody confirmed the direct binding of endogenous Trim21 and p21 proteins in myoblast cells (Fig. 5B), suggesting that Sugt1 may regulate p21 stability via Trim21. Consistently, significant p21 protein accumulation was detected in Sugt1 iKO cells cultured for 3 days as compared to Ctrl cells (Fig. 5C). The ternary interaction of Sugt1/Trim21/p21 was supported by subcellular co-localization detected by IF staining in C2C12 myoblasts (Fig. S4).

To further test whether Sugt1 can regulate ubiquitination of p21 protein in myoblast cells, C2C12 myoblasts were transfected with negative control siRNA (si-NC) or si-Sugt1 oligos and treated with proteasome inhibitor MG132. The total protein extraction was then subject to IP with an anti-p21 antibody. The result revealed that Sugt1 deficiency caused a dramatic decrease (by 72.4%) in the ubiquitination level of p21 thus the total p21 protein level was slightly increased (Fig. 5D), suggesting that Sugt1 can indeed mediate ubiquitination and degradation of p21 protein in myoblast cells.

To further demonstrate that Trim21 mediates Sugt1 function in regulating MuSC proliferation and cellular senescence, we found that knocking down Trim21 or Sugt1 by siRNA oligos resulted in a significant decrease (by 50.79% or 17.19%) in the percentage of EdU+ proliferating myoblasts compared to the control. Moreover, simultaneous knockdown of p21 and Trim21 fully rescued the reduced proliferation (Fig. 5E). Meanwhile, γH2AX staining revealed that Trim21 or Sugt1 knockdown significantly increased (by 132.6% and 31.6%) myoblast senescence and knocking down p21 could fully rescue the effect of Trim21 (Fig. 5E). Altogether, these results demonstrate that loss of Trim21 mediates the effect of Sugt1 in promoting myoblasts proliferation and preventing cellular senescence through binding and facilitating the ubiquitination and degradation of p21 protein.

## Discussion

In this study, we demonstrate an essential role of Sugt1 in MuSCs during muscle regeneration and aging. We found that specific inactivation of Sugt1 in MuSCs significantly impairs muscle regeneration by suppressing MuSC proliferation and cell cycle progression, consequently also causing MuSC cellular senescence. Moreover, during the natural aging process, Sugt1 inactivation aggravates muscle aging by impairing MuSC regenerative ability and increasing senescence. Mechanistically, by performing IP and MS we identified Sugt1 protein interactome and Trim21 protein as a bona fide binding partner. We further demonstrated that Trim21 binds with Sugt1 in myoblast cells to regulate the ubiquitination and degradation of p21 protein. Altogether, our findings uncover the previously uncharacterized functional role of Sugt1 in regulating MuSC proliferation and cellular senescence in muscle regeneration and aging.

In our prior study of lncRNA *SAM* function, Sugt1 is uncovered to be important for myoblast proliferation mainly using cultured C2C12 or MuSCs. We showed that Sugt1 is required for kinetochore assembly as loss of Sugt1 in myoblasts leads to typical defects associated with cell mitosis; for example, cells presented pronounced defects in kinetochore–microtubule attachment, spindle formation, and chromosome misalignments. Here in this study, we provide genetic evidence to confirm the essential role of Sugt1 in regulating myoblast proliferation. Furthermore, we uncover that the defective myoblasts with cell cycle arrest eventually undergo senescence with typical staining of SA-β-gal and histone γH2AX.

Considering the emerging interest in studying cellular senescence and muscle aging, we investigated the impact of Sugt1 loss on muscle aging and expectedly observed remarkable premature aging in the iKO mice. Long-term loss of Sugt1 resulted in a dramatic decrease in muscle strength and exercise performance. This presumably arises from the aggravated MuSC senescence, loss of the MuSC pool and their regenerative function. Transcriptomic analysis detected the increased production of SASP genes. In the future, it will be interesting to elucidate how these SASPs mediate cellular cross-talking and modulating niche environments thus contributing to muscle aging. It will also be possible to elucidate their potential to be used as serotherapeutic targets for treating sarcopenia condition.

**Figure 6.**
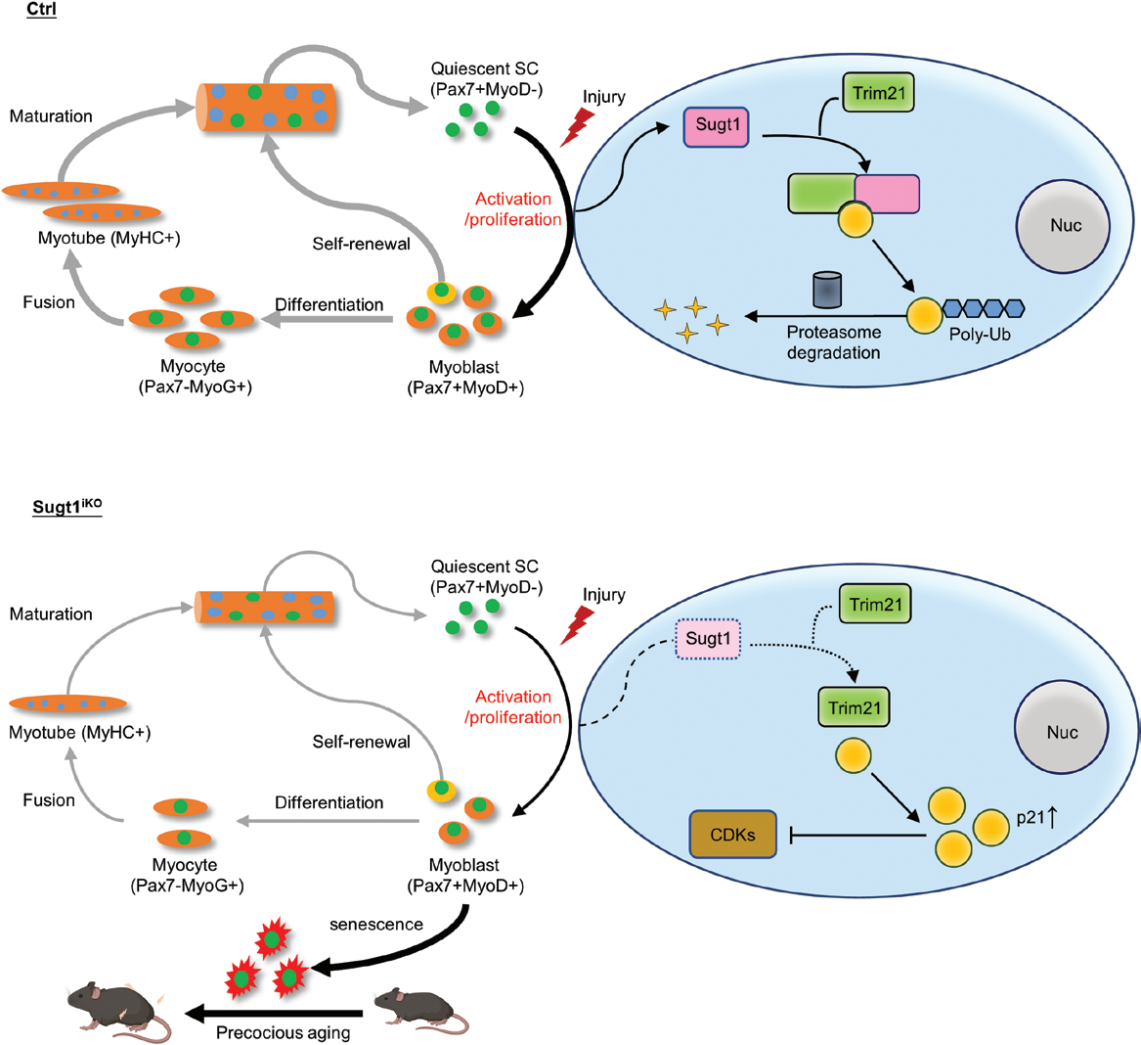
**A working model of Sugt1 function in MuSCs.**During acute injury-induced muscle regeneration, Sugt1 is induced upon SC activation and proliferation; it interacts with E3 ubiquitin ligase Trim21 to facilitate p21 ubiquitination and proteasome-driven degradation of p21, therefore driving the myoblast proliferation and completion of muscle regeneration. When Sugt1 is deleted in MuSCs, p21 accumulates due to decreased ubiquitination leading to cell cycle arrest and myoblasts senescence thus impairing regeneration. Long-term loss of Sugt1 during aging aggravates MuSC senescence and loss, causing premature muscle aging.

Mechanistically, since it is known that Sugt1 mainly functions in post-translational modification through protein–protein interaction [17, 34, 35], by identifying Sugt1 interacting proteins in C2C12 myoblasts, we obtain some insights into how Sugt1 regulates MuSCs lineage progression and cellular senescence. E3 ubiquitin ligase Trim21 was identified as a bona fide interacting partner of Sugt1 in myoblast cells. Sugt1 is known to interact with a conserved motif in the N-terminal of many E3 ubiquitin ligases through its TPR (tetratricopeptide repeat) domain, such as Skp1 of the SCF–Skp2 complex [36]. In addition, Sugt1 can also modulate the activity of these ligases by regulating their stability and function [19]. It is thus possible that Sugt1 can enhance the stability and activity of Trim21 E3 ligase, promoting the ubiquitination and degradation of Trim21 targets. Expectedly, as the known ubiquitination target of Trim21 [37], the protein level of the cellular senescence marker p21 increased in Sugt1 iKO cells while the ubiquitination of p21 was significantly decreased (Fig. 5C and 5D). We thus believe that Sugt1 regulates SC proliferation by interacting with Trim21 to mediate the degradation of p21 in a ubiquitin-dependent manner. Loss of Sugt1 in MuSCs leads to accumulation of p21, thus inhibiting the cell cycle regulator CDKs, which results in the cell cycle arrest, and stimulating precocious cellular senescence of MuSCs (Fig. 6). Altogether, our findings have defined a novel mechanism of Sugt1 in regulating MuSC proliferation and senescence during muscle regeneration and aging.

### Research limitations

More in-depth dissection of how Sugt1 modulates the function of Trim21 and how they regulate p21 will need to be conducted in the future. For example, sequential IPs can be performed to strengthen the presence of the ternary complex. Both genetic and biochemical assays can be performed for further mechanistic dissection.

## Methods

### Research ethics

All animal procedure ethics approval was granted by the Animal Experimentation Ethics Committee of the Chinese University of Hong Kong under Reference Number 19-008-MIS. All the mouse strains were housed either in the Laboratory Animal Services Centre (LASEC) of the Chinese University of Hong Kong or the LASEC Research Unit in the Prince of Wales Hospital with 12 h light/12 h dark cycles, temperature (22°C–24°C), humidity (40%–60%).

### Mice

Sugt1 flox mice (C57BL/6 background) were purchased from the Model Animal Research Center of Nanjing University (Nanjing, China). The *Pax7*^*CreER*^*/ROSA*^*EYFP*^ mice and *Pax7-nGFP* mice were generously gifted by Professor Zhenguo Wu (Hong Kong University of Science and Technology, HKUST). *Pax7*^*CreER*^ mice were ordered from the Jackson Laboratory. Sugt1 inducible knock-out strain was obtained by crossing *Sugt1*^*flox/flox*^ mice with *Pax7*^*CreER*^*/ROSA*^*EYFP*^ to obtain the Sugt1 iKO (*Sugt1*^*fl/fl*^; *Pax7*^*CreER/+*^; *ROSA*^*EYFP/+*^) and control (*Sugt1*^*+/+*^; *Pax7*^*CreER/+*^; *ROSA*^*EYFP/+*^). Inducible CreER (Estrogen Receptor)-mediated knockout of Sugt1 in adult mouse skeletal muscle stem cells was induced by intraperitoneal injection of Tamoxifen (TMX, T5648, Sigma) at 2 mg per 20 g body weight. The injection of TMX was injected for 5 days and, subsequently, one dose every 2 days until a total of seven doses injection. To induce acute skeletal muscle injury of the TA muscle, 50 µL of 1.2% BaCl_2_ (dissolved in sterile demineralized water) was intramuscularly injected into the TA muscle of adult mice. Muscles were dissected at day 3, 5, 7, and 14 post-injury. At least three pairs of littermates or same-age mice were sacrificed for all animal-related operations for collecting data. The sequence of genotyping primers is shown in Table S2.

### Muscle grip force measurement

A detailed protocol for grip strength tests of mice can be referred to Castro and Kuang [30]. The test involved placing a mouse on a metal grid so that all paws gripped a wire mesh grid attached to a force transducer. The mouse was then pulled by the tail with increasing force until it lost its grip. The force transducer displayed the maximum force attained. The measurement was done five times per session, and the final record of the mouse grip strength was the mean of the five measurements. The same person carried out all the tests. Mice were measured for body weight before the grip strength test and euthanized for analysis of muscle mass immediately after the final grip test.

### Treadmill exhaustion test

A detailed protocol for treadmill tests of mice can be referred to Castro and Kuang [30]. All mice were exposed to treadmill exercise (10 cm/s for 5 min, inclination set at +5°) for a familiarization period of 3 consecutive days before the exercise endurance test. Performed a treadmill exhaustion test for the mice the day after the last training. During the test, the exercise started at 10 cm/s for 5 min with +5° inclination. Then gradually increased the speed with +2 cm/s every 2 min. The mouse was forced to run until exhaustion and the total running time and distance was recorded.

### Cell culture

Mouse C2C12 myoblast cells (CRL-1772) were obtained from American Type Culture Collection (ATCC) and cultured in DMEM medium (12800-017, Gibco) with 10% fetal bovine serum (FBS, 10270-106, Gibco) and 1% penicillin/streptomycin (P/S, 15140-122, Gibco) at 37°C in 5% CO_2_ incubator. FACS-sorted MuSCs were cultured in Ham’s F10 medium supplemented with 20% FBS, 5 ng/mL b-FGF (PHG0026, ThermoFisher Scientiﬁc) and 1% P/S at 37°C in 5% CO_2_ incubator.

### Single myofibers isolation and culture

Single myoﬁbers were isolated as previously described before according to Chen et al. [38] Brieﬂy, each extensor digitorum longus muscle was excised from tendon to tendon and digested in F10 medium containing Collagenase type II (800 U/mL) at 37°C in the water bath for 75 min. A large bore Pasteur pipette was used to release myoﬁbers in the warm medium by ﬂushing several times until enough single myoﬁber was observed. Live single myoﬁbers were then transferred to a new dish for removing dead ﬁbers and debris. Isolated single myoﬁbers were cultured in F10 medium supplemented with 10% heat-inactivated horse serum (HIHS, 26050088, Gibco) and 1% P/S.

### Satellite cell isolation and culture

Mouse MuSCs were isolated from 2-month-old *Pax7-nGFP* mouse as previously described according to Li et al. [24]. Brieﬂy, the hindlimb muscles were collected and minced followed by digestion in 10 mL Ham’s F10 medium (N6635, Sigma) with Collagenase type II (LS004177, Worthington, 1000 U/mL) for 90 min immersed in a shaking water bath at 37°C. Then, the muscles were washed with washing buffer (Ham’s F10 medium supplemented with 10% HIHS) and further digested in a washing medium containing Collagenase II (800 U/mL) and Dispase (11 U/mL) for 30 min in the water bath at 37°C. The mixture was further isolated by passing through a 20 G needle and ﬁltered through a 40-mm cell strainer. MuSCs were washed once before being sorted out by BD FACSAria (BD Biosciences) based on GFP reporter protein.

### EdU incorporation

EdU incorporation assay was performed as described previously according to So et al. [39]. Edu assay was performed following the manufacturer’s instructions (C10339, ThermoScientific). Cells on coverslips were incubated in a culture medium with 10 mM EdU for designated times. Cells were then washed three times in PBS and fixed with 4% PFA for 10 min. EdU+ cells were labeled using “click” chemistry with an Alexa Fluor 594-conjugated azide, and DAPI (P36931, Life Technologies) was mounted for staining the cell nucleus. Pictures were captured with a fluorescence microscope (Leica).

### Cell-cycle analysis by FACS

C2C12 myoblasts or ASCs were synchronized to the mitotic stage by nocodazole treatment for 3 h or 6 h before ﬁxation with 5 mL pre-cold 70% ethanol at 4°C overnight. The cells were washed once with PBS to remove residues, 300 mL of propidium iodide (PI) solution and 20 mL of 10 mg/mL RNase was supplemented and incubated at 37°C for 45 min (protecting from the light), the cells were then transferred to FACS tubes for FACS analysis. To isolate living C2C12 myoblasts at different cell cycle stages, two drops of Hoechst 33342 (ThermoFisher R37165) were added per 1 mL cell culture medium and the cells were incubated for 30 min at 37°C before subjecting to FACS sorting.

### Cellular senescence SA-β-gal detection staining

Cellular senescence was evaluated by β-galactosidase activity using β-galactosidase Senescence Kit (#9860, Cell Signaling Technology). Brieﬂy, cells were ﬁxed for 15 min followed by washing in PBS twice. Then, ﬁxed cells were incubated with β-galactosidase staining solution at 37°C in a dry incubator (no CO_2_) at least overnight. The cells were then observed under a microscope for the development of blue color.

### Total RNA extraction and real-time qPCR

Total RNAs were extracted using TRIzol reagent according to the manufacturer’s instructions. Isolated RNAs were reversely transcribed to complementary DNA (cDNA) by using HiScript II Reverse Transcriptase Kit (R211, Vazyme), quantitative mRNA expression was performed with SYBR Green Master Mix (4368708, Applied Biosystem) and LightCycler 480 Real-time PCR system, 18S rRNA was used for normalization. Primers for qRT-PCR were listed in Table S2.

### Western blotting

Cell lysates were collected by direct lysis with RIPA buffer (50 mM Tris–HCl, pH 7.5, 150 mM NaCl, 1.0 mM EDTA, 0.1% SDS, 1% Sodium deoxycholate, and 1% Triton X-100) supplemented with protease inhibitor cocktail (PIC, 88266, Thermo Fisher Scientiﬁc), and samples were boiled to denature at 100°C for 10 min before SDS–PAGE gel electrophoresis. The proteins were wet-transferred to PVDF Western blotting membrane (03010040001, Roche) after electrophoresis, membranes were blocked for 1 h with 5% milk in TBST before overnight incubation with below primary antibodies: mouse anti-Sugt1 (1:500, sc-81822, Santa Cruz), mouse anti-α-Tubulin (1:5000, B-5-1-2, Santa Cruz), mouse anti-Flag (1:1000, F1804, Sigma), mouse anti-Ub (1:5000, sc-8017, Santa Cruz), mouse anti-Trim21 (1:500, PA5-120224, Thermo Fisher Scientific), mouse anti-p21 (1:500, sc-6246, Santa Cruz), and mouse anti-p16 (1:500, sc-1661, Santa Cruz). Membranes were then washed three times and incubated with horseradish peroxidase (HRP)-conjugated secondary mouse anti-rabbit antibody (sc-2357, Santa Cruz Biotechnology) or m-IgGκ BP-HRP antibody (sc-516102, Santa Cruz Biotechnology) for 1 h at RT. Reactive proteins were detected by Enhanced Chemiluminescence (ECL) reagent (K-12045-D20, Advansta).

### Cell fractionation

C2C12 cells at MB or MT stage were collected in cold PBS, washed twice, and then incubated in buffer A (HEPES–KOH 50 mM, pH 7.5, 10 mM KCl, 350 mM sucrose, 1 mM EDTA, 1 mM DTT, 0.1% Triton X-100) for 10 min on ice with occasional shaking. The nuclei were harvested by brief centrifugation (2000 *g*, 5 min). The supernatant was collected as the cytoplasmic fraction. The nuclei were further washed twice with buffer A without Triton X-100. Protein extraction and Western blotting of cytoplasmic and nuclear fractions were performed as previously described according to Zhao et al. [40].

### *In vivo* ubiquitination assay

C2C12 cells were transfected with flag-ubiquitin plasmids and si-Trim21 or si-Sugt1 oligos. MG132 (10 µM) was applied to cells for 4 h at 1.5 days after transfection. The cells were then lysis with lysis buffer and proceeded to immunoprecipitation with the p21 antibody. The ubiquitination levels of protein were then detected by immunoblotting with Ub antibody and measured by using Imag J (NIH).

### Immunoprecipitation assays

Co-IP of C2C12 cells was performed as previously described according to Qiao et al. [41]. Cells were lysed with lysis buffer (50 mM Tris–HCl, pH 8.0, 150 mM NaCl, 0.1% SDS, 0.5% sodium deoxycholate, and 1% NP-40). Equal amounts of protein lysates from different samples were incubated with 10 µg of antibody overnight at 4°C with rotation. After incubation, the mixture was incubated with Dynabeads™ Protein G (Invitrogen) for 6 h at 4°C. For the Flag-Sugt1 expressing cell lysates, total lysates were directly incubated with 10 µL Anti-Flag Magnetic Beads (HY-K0207, MedChem Express) at 4°C overnight. The immunocomplexes were washed with washing buffer (10 mM Tris–HCl, pH 7.5, 150 mM NaCl, 1.0 mM EDTA, 1.0 mM EGTA, and 1% Triton X–100) four times. Finally, the immunocomplexes were boiled with emerging in 2× loading buffer applied to SDS–PAGE or LC/MS analysis.

### Mass spectrometry

The band uniquely present in the SAM pull-done lane after Coomassie blue staining was cut out and subjected to LC–MS/MS analysis (Shanghai Applied Protein Technology, Shanghai, China). The MS scan was performed with the following parameters: positive ion detection; scan range (m/z) = 300–1800; resolution = 70,000 at 200 m/z automatic gain control (AGC) target = 1e6; maximum injection time = 50 ms; dynamic exclusion = 60 s. polypeptide and poly-peptide fragments were collected according to the following parameters: after each full scan, 10 fragment maps (MS2 scan) were collected, MS2 activation type was HCD, isolation window was 2 m/z, second-level mass spectral resolution was 17,500 at 200 m/z, collision energy was 30 eV, and underﬁll was 0.1%. The MS/MS spectra were searched with MASCOT engine (Matrix Science, version 2.2). The following option was used: peptide mass tolerance = 20 ppm, fragment mass tolerance = 0.1 Da, enzyme = trypsin, max missed cleavages = 2, ﬁxed modiﬁcation: carbamidomethyl (C), and variable modiﬁcation: oxidation (M), acetyl (protein N-term). The identiﬁed proteins were retrieved from the uniport mouse database (ref. no. 73952; download time: 20130313). Ion score ≥ 20. The number of unique peptides (Unique PepCount) and cover percent (Cover%: the number of detected amino acids/total number of amino acids in the protein) were used to identify proteins. In this study, one sample was analyzed once by LC–MS/MS.

### Hematoxylin-eosin staining

Hematoxylin-eosin (H&E) staining on cryo-sections was performed as previously described according to Diao et al. [42]. The frozen slides were transferred to RT for equilibration and immersed in hematoxylin for 7 min. The eosin was used to stain the slides for 45 s followed by dehydration and transparency. Lastly, the slides were mounted with polystyrene before observation.

### Immunofluorescence staining

For IF staining on cells, cells were ﬁxed on coverslips with 4% PFA and permeabilized with fresh 0.5% NP-40 (diluted with PBS) for 10 min. Following by blocking with 3% BSA and incubation with designated primary antibodies overnight at 4°C. The cells were washed with PBS three times and incubated with secondary antibodies for 1 h at RT (protected from light). Then, the cells were mounted with DAPI solution before observation. IF staining on TA sections was performed as before [43], mouse TA muscles were dehydrated in 30% sucrose for 1 h at RT and embedded with optimal cutting temperature compound (O.C.T, 4583, SAKURA), the tissue blocks were stored at −80°C before cutting into 5 mm sections. Tissue slides were ﬁxed and permeabilized before antigen retrieval with 0.01 M pH 6.0 citric acid in a high-heated microwave, followed by blocking in 4% bovine serum albumin (BSA, IgG-Free, Protease-Free) (001-000-162, Jackson ImmunoResearch) for 3 h. Then, the endogenous mouse IgG was blocked by incubation with AfﬁniPure Fab Fragment Goat Anti-Mouse IgG (H + L) (115-007-003, Jackson ImmunoResearch). Staining with the primary antibody omitted was used as the negative control. The appropriate primary antibodies were used as follows: mouse anti-MyoD (Dako MG3512; 1:200); mouse anti-eMyHC (NCL-MHC-d, Leica; 1:200); rabbit anti-laminin (Sigma L9393; 1:800); Pax7 (Developmental Studies Hybridoma Bank; 1:50); MF20 (Developmental Studies Hybridoma Bank; 1:50); Myogenin (Santa Cruz sc-576; 1:200); γH2AX (Santa Cruz sc-517336; 1:200); anti-p21 Monoclonal Antibody (ThermoFisher MA514949; 1:50); TRIM21 (ThermoFisher PA518147; 1:50); Sugt1 (Santa Cruz sc-81822; 1:100); all ﬂuorescent images were captured with a fluorescence microscope (Leica) and confocal microscope (ZEISS LSM 9).

### RNA sequencing and data analysis

RNA-Seq of freshly isolated MuSCs was performed by BGI. TRIzol reagent (Invitrogen, 15596018) was used to extract total RNAs from Ctrl and iKO MuSCs. DNA libraries were generated from polyA+ purified mRNA samples. Bioanalyzer was used to examine the library’s size and purity, and real-time PCR was used to detect the quantity and concentration. Data from sequenced fragments were mapped to mouse genome mm10 using TopHat. The abundance levels of transcripts were determined by cufflinks in the form of FPKM. GO analysis was performed by the online bioinformatics resource DAVID. Gene set enrichment analysis (GSEA) was performed by the GSEA and MSigDB software from UC San Diego and Broad Institute.

### Statistical analysis

All data were present as mean ± SD for at least three individual replicates. The statistical significance between separate groups was calculated with Student’s *t* test and one-way ANOVA. Significance was described as * represents *P* < 0.05, ** represents *P* < 0.01, and *** represents *P* < 0.001.

## Data availability

The authors confirm that the data supporting the findings of this study are available within the article and its supplementary materials. Raw data that support the findings of this study are available from the corresponding author, upon request.

## Supplementary Material

lnad039_suppl_Supplementary_Materials

lnad039_suppl_Supplementary_Table_S1

lnad039_suppl_Supplementary_Table_S2
