# Supplementary material for: Sugt1 loss in skeletal muscle stem cells impairs muscle regeneration and causes premature muscle aging
Source: Life Med. 2023 Nov 2;2(4):lnad039. doi: 10.1093/lifemedi/lnad039 (PMC11749260; doi:10.1093/lifemedi/lnad039)

Supplementary Materials

Sugt1 Loss in Skeletal Muscle Stem Cells Impairs Muscle Regeneration and Causes Premature Muscle Aging

Supplementary Figures

Figure S1. Sugt1 subcellular localization and Sugt1 iKO mouse morphology. A. Cellular fractionation was performed in C2C12 myoblasts, and the subcellular localization of *Sugt1* was detected by Western blotting. α-tubulin and Histon-H3 was used as a control. **B.** The expression levels of Sugt1 proteins and p21 proteins during cell cycle were determined by Western blotting in C2C12 myoblasts. β-Actin staining was used as the loading control. C. Images of 3 pairs of 2-month-old Ctrl and iKO mice. **D.** Quantification of body weight of 2-month-old Ctrl and iKO mice. n=3 mice. **D.** H&E staining of the TA muscle sections from the above mice.

Figure S2. Sugt1 deletion impairs MuSC activation and differentiation. **A**. Left: C2C12 myoblasts were transfected with a vector or Flag-tagged Sugt1 overexpression plasmid. EdU incorporation assay was performed and IF staining for EdU was performed at 48h post-transfection. Scale bar: 50 µm. Right: Right: Quantification of the percentage of EdU+ cells. n=5. **B.** Left: SCs from Ctrl and iKO mice were cultured for 24 h with EdU before IF staining of Pax7 and EdU. EdU incorporation assay was performed and IF staining for Pax7 and EdU was performed in ASC-24h. Scale bar: 50 µm. Right: Quantification of the percentage of Pax7+EdU+ cells. n=3 mice. **C.** Left: IF staining for Pax7 and MyoD was performed in ASCs isolated from Ctrl or iKO mice and cultured for 24h; Scale bar: 50 µm. Right: Quantification of the percentage of Pax7+MyoD+ cells. n=3 mice. **D.** Left: single myofibers were isolated from Ctrl or iKO mice and IF staining for Pax7 and MyoD was performed at 24h. Scale

bar: 50 μm; Right: Quantification of the number of Pax7+MyoD+ cells per myofiber. n=3 mice, >30 myofibers were counted for each mouse. **E**. Left: IF staining for MyoG and MyoD was performed in ASCs isolated from Ctrl or iKO mice and cultured for 48h; Scale bar: 50 μm. Right: Quantification of the percentage of MyoG+MyoD+ cells. n=3 mice. **F.** Left: single myofibers were isolated from Ctrl or iKO mice and IF staining for MyoG and MyoD was performed at 72h Scale bar: 50 μm; Right: Quantification of the number of MyoG+MyoD+ cells per myofiber. n=3 mice, >30 myofibers were counted for each mouse. **G.** Left: 20 staining in 4 days cultured SCs isolated from Ctrl and iKO mice. Scale bar: 50 μm. Right: Quantification of the fusion index of myotubes (≥2 nuclei)/total MF20+ cells). DM differentiation medium.

Figure S3. Transcriptomic profiling by RNA-seq in aged Ctrl and Sugt1 iKO mice. A. RNA- seq was performed in ASC-48h from 24-month-old iKO and Ctrl mice. Volcano plot showing the down- and up-regulated genes in 24-month-old iKO vs. Ctrl. B. Expression fold of *p16* mRNA (FPKM) from the above RNA-seq data. **C.** Gene Set Enrichment Analysis (GSEA) for the SASP gene set.

Figure S4. Subcellular co-localization of Sugt1, Trim21 and p21. A. Co-staining of Sugt1, Trim21, and p21 proteins in C2C12 myoblast cells by IF. Images were taken using ZEISS Confocal Microscope LSM9. Scale bar: 5 μm.

Supplemental Table caption

Table S1. Transcriptomic profiling in Sugt1 iKO SCs from young and aged mice.

Table S2. Information on oligonucleotides and primers used in the study.

Supplementary Figure S1

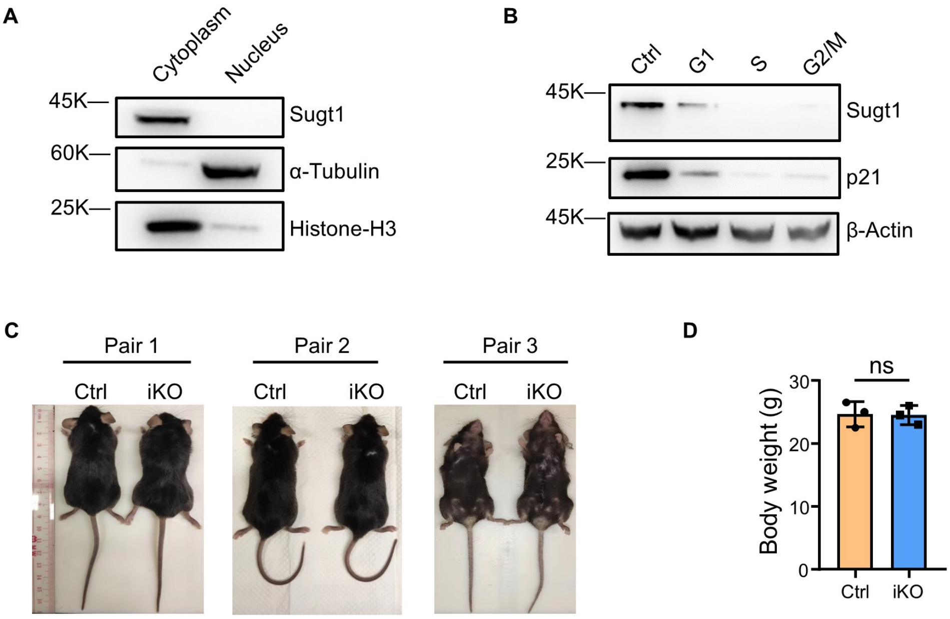

Supplementary Figure S2

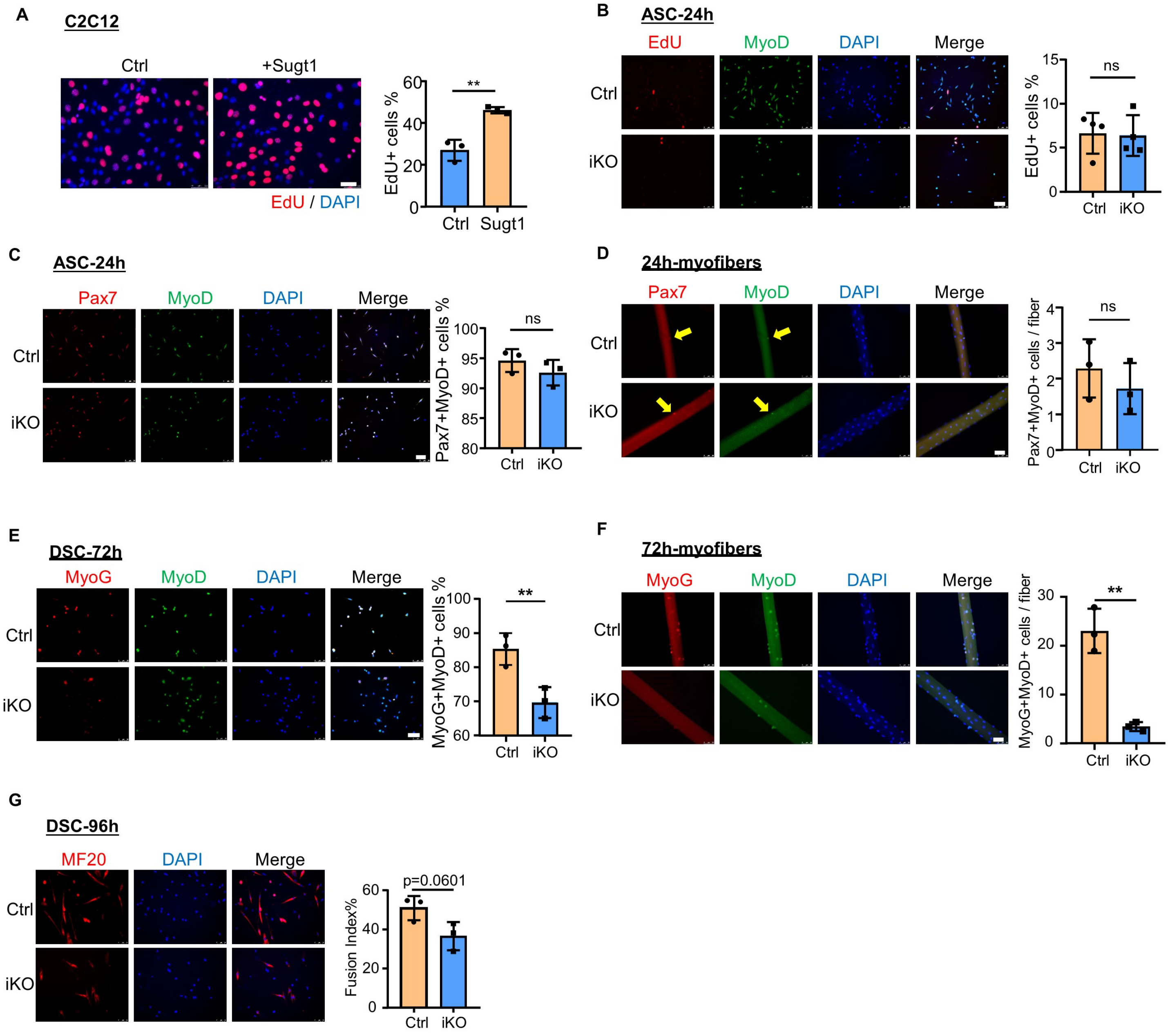

Supplementary Figure S3

A <u>**RNA-seq: aged ASC**</u>

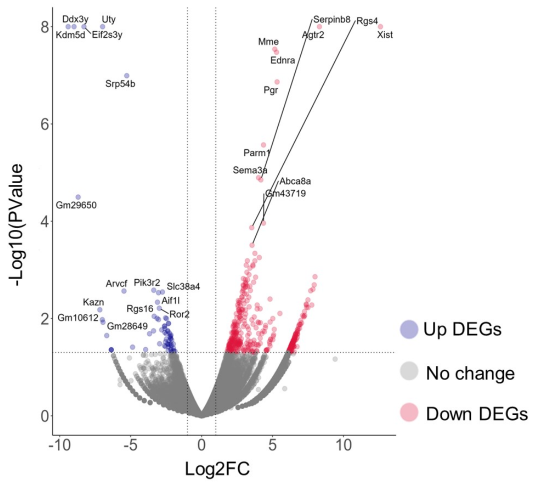

B

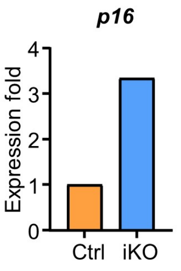

C

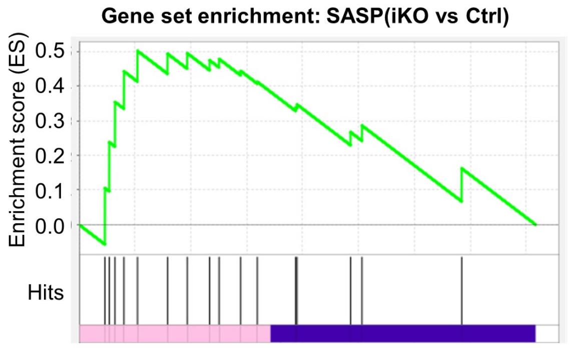

Supplementary Figure S4

A

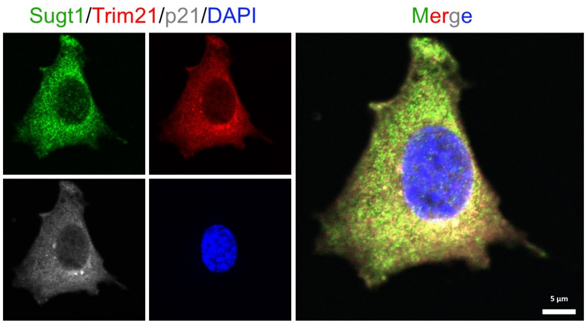

Supplement: lnad039_suppl_Supplementary_Materials [file lnad039_suppl_Supplementary_Materials.pdf]
